# Isolated Transverse Sacrum Fracture: A Case Report

**DOI:** 10.1155/2011/741570

**Published:** 2011-06-23

**Authors:** Cemil Kavalci, Gökhan Akdur, Mustafa Burak Sayhan, Ozgur Sogut, Mehmet Tahir Gökdemir

**Affiliations:** ^1^Department of Emergency Medicine, Faculty of Medicine, Trakya University, Edirne, Turkey; ^2^Department of Emergency Medicine, Faculty of Medicine, Harran University, Sanliurfa, Turkey

## Abstract

Sacral fracture commonly results from high-energy trauma. Most insufficiency fractures of the sacrum are seen in women after the age of 70. Fractures of the sacrum are rare and generally combined with a concomitant pelvic fracture. Transverse sacral fractures are even less frequent which constitute only 3–5% of all sacral fractures. This type of fractures provide a diagnostic challenge. We report a unique case of isolated transverse fracture of sacrum in a young man sustained low-energy trauma. The patient presented to our emergency department after several hours of injury, and diagnosed by clinical features and roentgenogram findings.

## 1. Introduction

Reports of transverse fractures of the sacrum in the literature are not common. This issue has been attributed mostly to the challenge of obtaining diagnostic roentgenograms but also to the fact that this type of a fracture rarely is suspected [1]. A longitudinal fracture of the sacrum can be associated with approximately 45% of pelvic fractures. However, a transverse fracture of the sacrum is even less frequent, and accounted for only 4.5% of sacral fractures in humans [1, 2]. Lower sacral transverse fracture are often due to direct traumatic force against coccyx, and it is related to fall and resulting in break at the kyphos of sacrum mostly through the body of the lower 4-5 sacral vertebrae. Rarely, neurological deficit may accompany these fractures [2, 3]. There is often a delay in definitive diagnosis, if the quality of the roentgenograms is not adequate and if they are not examined specifically for the lesion. Most neurological insults associated with delays in diagnosis [1, 3]. Here, a case of lower fourth sacral vertebrae fracture associated with low-energy fall, who was diagnosed by clinical suspicion and appropriate roentgenogram, is reported.

## 2. Case Report

**Figure 1:**
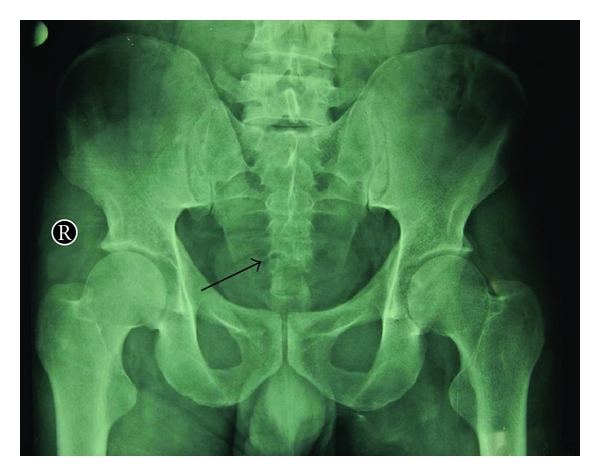
The anteroposterior roentgenogram of the lumbosacral area demonstrating a transverse fracture line at the right and anterior aspect of the fourth sacral vertebrae.

A 37-year-old male was admitted to the emergency department (ED) with low back pain and tenderness following injury. He fell on his back onto a rough surface three hours ago. On examination, there were no weakness or anaesthesia in both the lower limbs, and inability to void urine, and to control bowel function. Roentgenogram of the pelvis revealed irregularity in the arcuate lines of the lower two sacral foramina associated with a transverse sacrum fracture through fourth sacral vertebrae (Figure 1). The fracture line from the anterior to the posterior aspect of the fourth sacral segment without narrowing of the sacral canal was confirmed later by lumbosacral tomography. As the patient had no neurological deficit, he was discharged home on the same day with recommendations for bed rest and analgesics. 

## 3. Discussion

Isolated fractures of the sacrum are rare and generally these occur in combination with pelvic rim fractures [2]. Sacral fracture commonly results from high-energy trauma. Isolated sacral fractures which occur by shear forces on the pelvic ring are seen less commonly, and they are commonly transversely oriented [4]. Most fractures of the sacrum occur in women (94,3%) of advanced age (mean age : 70,6 years). A complete and careful physical and neurological examination will contribute a definitive diagnosis in suspecting this rare injury. Fracture of the sacrum should be suspected in the presence of low back or sacral pain and tenderness [5]. In addition, depending on the degree of root involvement, there may be hypesthesia or segment provides sensation to the posterior aspect of the thigh, the posterolateral aspect of the calf, and the sole of the foot. The third sacral segment involves not only the superior medial aspect of the thigh but also part of the sacral region. The perianal region, anus, penis, posterior part of the scrotum, and posterior portion of the labia majora are innervated through the fourth and fifth sacral-nerve roots. These are the areas that must be tested carefully to elicit evidence of a sacral fracture [1, 5]. In the present case, despite he was young man and sustained low-energy trauma due to fall on a ground, the injury led to a stable sacral fracture associated with lower back pain and sacral tenderness. Transverse sacral fractures have been classified as upper and lower fractures. Lower sacral transverse fracture are often resulting from direct traumatic force. Rarely, it can produce neurological damage [1]. The muscles of the lower limb are supplied by multiple peripheral nerve roots, predominantly above the second sacral level. Therefore, no extensive paralysis will be developed in patients who have only a sacral fracture, as in the present case. If such paralysis does exist, other associated injuries should be suspected. Some of the patients with transverse sacral fractures demonstrates a neurological deficit of importance which mainly concerns the bowel or bladder [1, 2]. Furthermore, attention should always be paid to the bladder because the neurological deficit may not be apparent immediately after the injury Lumbalgia, pain of the lower limbs, functional disability of the bladder, and bowel, seem due to a narrowed lumbar canal, a disc-nervous root conflict or a vertebral fracture [2]. No neurological deficit in the present case was demonstrated on arrival and during the 12-hour observation in the ED. As described by Rowell [6], transverse sacral fractures almost always involve the lower three segments of the sacrum (S3–S5). The ligamentous support of this region is achieved by the sacroiliac ligament, the sacrospinous ligament, and, more importantly, the sacrotuberous ligament. The coccyx may act as a lever arm on the body of the sacrum. The force so applied is resisted primarily by the ligamentous structures just mentioned. In the present case, the fractured fourth sacral vertebrae, injured by either of the mechanisms pointed out above, was driven forward, resulting in injury to the sacral region [1, 6]. Diagnosis is often late, or sometimes is not even made. Standard roentgenograms of lumbosacral region with an adequate quality can able to predict ordinary degenerative lesions or fracture displacement narrowing the sacral canal in every case, and seem sufficient to make a definitive diagnosis of sacral fractures. If the quality of the roentgenograms is not adequate, fractures of sacrum are discreet, without displacement, often hidden by gas, stercoral stasis, or vascular calcifications. In this setting, a computed tomography of the lumbosacral region is always mandatory to confirm the diagnosis [1]. Treatment of transverse sacral fractures uncomplicated by neurological deficits should be conservative. However, in case of sacral root injuries with displaced sacrum fracture, decompression such as gibbectomy is recommended [1, 2, 7]. Outcomes of operative decompression are debatable, and even conservative treatment has been advocated [2].

## 4. Conclusion

Because isolated transverse sacrum fractures are rarely seen, and it can be a challenge of obtaining appropriate roentgenograms, the early diagnosis might be overlooked. The ED physician should be suspected of this type of fractures in the presence of low back or sacral pain and tenderness. Conventional roentgenograms can allow for visualization of a transverse sacral fracture, if the quality of the roentgenograms is adequate. In most instances, treatment is consisted of analgesia, sedation, and bed rest.
